# Clinical importance of the superficial temporal artery in neurovascular diseases: A PRISMA-compliant systematic review

**DOI:** 10.7150/ijms.36698

**Published:** 2019-09-20

**Authors:** Kun Hou, Yunbao Guo, Kan Xu, Jinlu Yu

**Affiliations:** Department of Neurosurgery, The First Hospital of Jilin University, Changchun, 130021, China.

**Keywords:** Superficial temporal artery, aneurysm, arteriovenous fistula, bypass

## Abstract

The superficial temporal artery (STA) plays a very important role in neurovascular diseases and procedures. However, until now, no comprehensive review of the role of STA in neurovascular diseases from a neurosurgical perspective has ever been published. To review research on the clinical importance of STA in neurovascular diseases, a literature search was performed using the PubMed database. Articles were screened for suitability and data relevance. This paper was organized following the Preferred Reporting Items for Systematic Reviews and Meta-Analyses (PRISMA) guidelines. According to the literature, STA is one of the terminal branches of the external carotid artery and can give off scalp, muscle, and transosseous branches. STA-middle cerebral artery (MCA) bypass is very useful for intracranial ischemic diseases, including moyamoya disease, chronic ICA and MCA insufficiency, and even acute ischemic stroke. For intracranial complex aneurysms, STA bypass remains a major option that can serve as flow replacement bypass during aneurysmal trapping or insurance bypass during temporary parent artery occlusion. Occasionally, the STA can also be involved in dural AVFs (DAVFs) via to its transosseous branches. In addition, the STA can be used as an intraoperative angiography path and the path to provide endovascular treatments. Therefore, STA is a very important artery in neurovascular diseases.

## Introduction

The superficial temporal artery (STA) is one of the terminal branches of the external carotid artery (ECA), and it together with other branches of the ECA, supplies the face and scalp 1. Currently, the STA plays a very important role in neurovascular diseases and procedures, such as bypass for intracranial ischemic diseases, bypass for intracranial complex aneurysms, STA aneurysms, direct arteriovenous fistulas (AVFs), dural arteriovenous fistulas (DAVFs), and intraoperative angiography and endovascular treatments (EVTs) 2-7.

Therefore, a comprehensive understanding of the role of STA in neurovascular diseases is important for multiple disciplines 8. However, until now, no comprehensive review of the clinical importance of STA in neurovascular diseases from a neurosurgical perspective has ever been published. Hence, in this paper, a literature search was performed using the PubMed database and relevant search terms. This article was organized following the Preferred Reporting Items for Systematic Reviews and Meta-Analyses (PRISMA) guidelines and was established as a systematic review 9.

## Materials and methods

This systematic review was conducted in accordance with the PRISMA guidelines 9. Eligible English language articles (case reports, case series, and studies of the STA in neurovascular diseases) were identified through searches of PubMed publications (last search date was May 13^th^ 2019).

**Figure 1:**
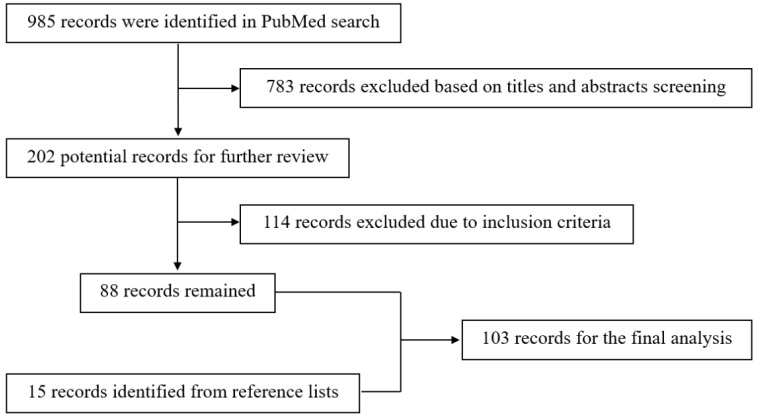
Flow chart of the search strategy.

The search algorithm used the terms “superficial temporal artery,” “ischemic disease”, “moyamoya disease”, “aneurysm”, and “arteriovenous fistula” as key words in relevant combinations. The reference lists of the identified articles were also manually searched for additional studies. The resulting flow chart is shown in Figure 1.

The inclusion criteria were as follows: a) full text was available, b) clinical data were complete, and c) STA was involved or used in all of the cases in these articles. The studies without sufficient descriptions of the STA role were excluded.

## Results and discussion

After a review of the obtained literature, the current status of the role of STA in neurovascular diseases was summarized in terms of the anatomy of STA, bypass for intracranial ischemic diseases, bypass for intracranial complex aneurysms, STA aneurysm, direct AVF, DAVF, and intraoperative angiography and EVTs.

### (1). Applied anatomy of the STA

The STA originates from the ECA deep to the superficial pole of the parotid and ascends approximately 1 cm anterior to the auditory canal 10. It courses over the root of the zygoma, and it then divides into the frontal and parietal branches approximately 2 to 3 cm superior to the zygoma 11. The STA runs with the superficial temporal vein (STV) in a wavy fashion 12.

The frontal branch of the STA supplies the skin and muscles of the forehead and anastomoses with the supraorbital and supratrochlear arteries, and the parietal branch supplies the temporal and parietal regions 13-15. The STA forms numerous anastomoses with the middle and deep temporal artery and gives off some transosseous branches 6, 14, 16.

The STA has a suitable length and diameter 17. In a cadaveric study by Pinar et al. (2006), the mean diameter of the STA at the zygomatic arch was determined to be 2.7 mm 18. In another study by Medved et al. (2015) based on digital subtraction angiography (DSA), the surgically average “working lengths” of the frontal and parietal branches above the upper margin of the zygoma were 106.4 mm and 99.7 mm, respectively 19.

**Figure 2:**
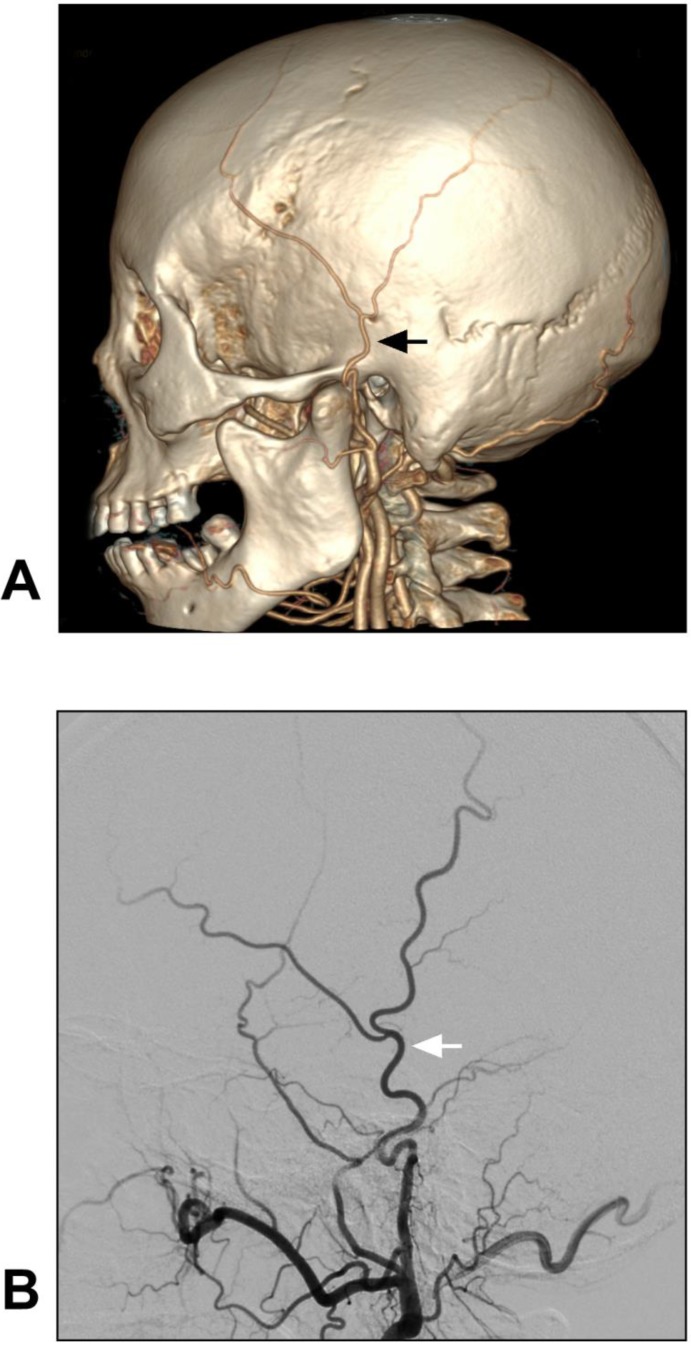
** Anatomy of STA on CTA and DSA.** A-B: CTA (A) and DSA images of the ECA (B) show the STA courses over the root of the zygoma before it roughly divides into the frontal and parietal branches. The STA is indicated by black and white arrows in A and B respectively. These CTA and DSA images were obtained from different patients. **Abbreviations:** CTA: computed tomography angiography; DSA: digital subtraction angiography; ECA: external carotid artery; STA: superficial temporal artery.

The anatomy of the STA can be evaluated via computed tomographic angiography (CTA) and DSA 20. The anatomical angiography of the STA is shown in Figure 2.

### (2). Bypass for intracranial ischemic diseases

#### (i) Moyamoya disease

Moyamoya disease (MMD) is characterized by progressive stenosis or occlusion of the terminal portion of the bilateral internal carotid arteries (ICAs) with extensive and abnormal moyamoya-like collaterals in the brain base 21. In MMD, there is a risk of ischemic stroke due to hemodynamic insufficiency and intracranial hemorrhage due to fragile collaterals 22.

Currently, the strongest agreement regarding treatment for symptomatic MMD in the chronic phase is extracranial-intracranial (EC-IC) bypass, including direct bypass and indirect encephaloduroarteriosynangiosis 23-25.

Indirect procedures can result in excellent results in children with MMD and have therefore been widely used 26, 27. In adult MMD, the combination of direct and indirect methods is beneficial as direct surgery can establish immediate blood flow, while indirect procedures can lead to a prolonged increase in perfusion 28, 29. In some patients, direct bypass can lead to spontaneous disappearance of the aneurysm in the collaterals 30. Therefore, direct bypass plays a very important role in MMD treatment.

Direct EC-IC bypass includes STA-middle cerebral artery (MCA), STA-anterior cerebral artery (ACA), STA-posterior cerebral artery (PCA), and occipital artery (OA)-PCA anastomoses, among which STA-MCA bypass is the most common direct revascularization procedure. It is mainly used to address the MCA territory but also supports the ACA territory via leptomeningeal anastomoses 31-34.

STA-MCA bypass is typically performed using only a single STA donor branch to perform a single anastomosis. Recently, single-vessel double anastomosis and double-barrel STA-MCA bypass appeared 35-37. These new techniques are planned to enhance STA-MCA flow capacity, but their effects as a surgical treatment for MMD remains controversial 38, 39.

**Figure 3:**
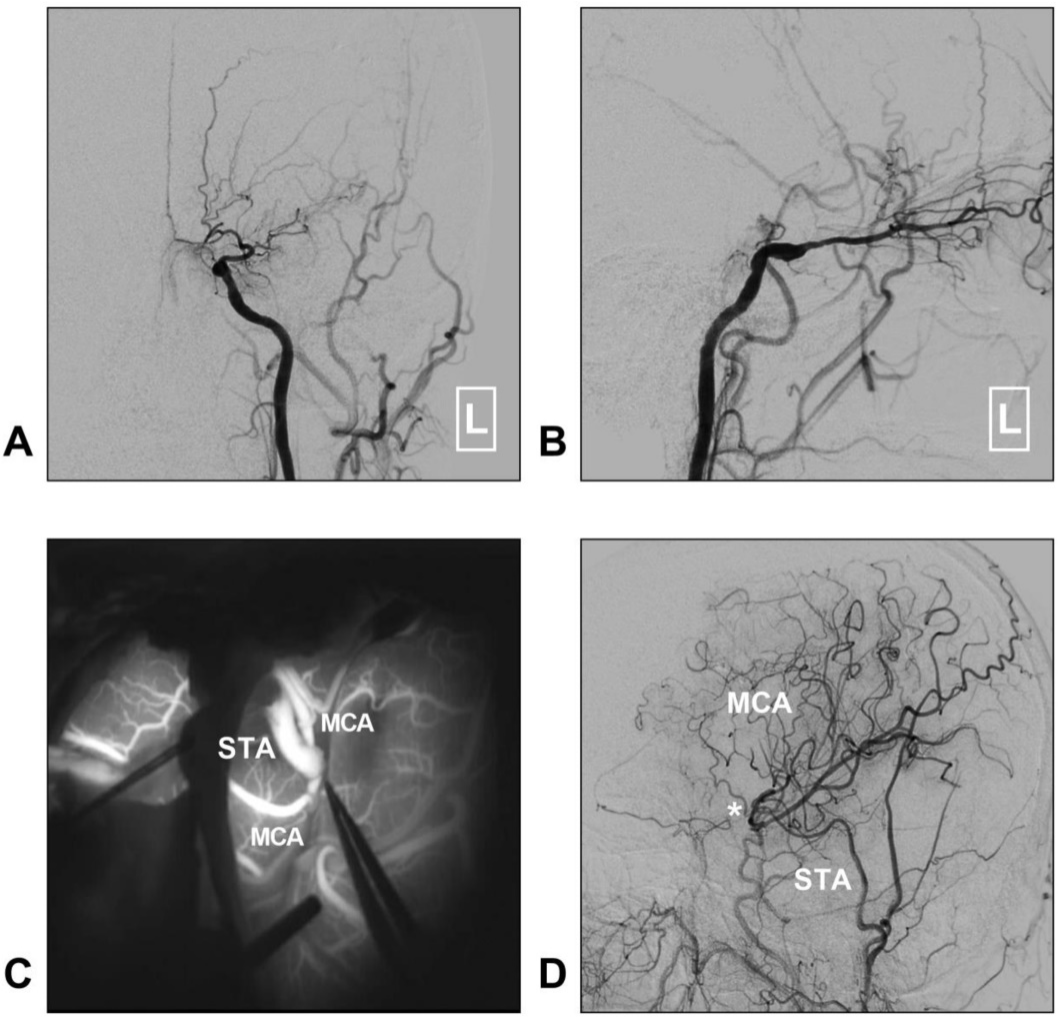
** STA-MCA bypass for MMD.** A-B: Left CCA angiogram shows steno-occlusive alteration of the ICA terminal; the ophthalmic artery is preserved. C: Intraoperative indocyanine green angiography shows that an STA-MCA bypass is established. D: Follow-up DSA shows that the distal MCA is reconstructed. The asterisk indicates the anastomosis point. **Abbreviations: C**CA: common carotid artery; DSA: digital subtraction angiography; MCA: middle cerebral artery; MMD: moyamoya disease; STA: superficial temporal artery.

Although STA-MCA bypass has been described as a low-flow system, it is sufficient for MMD 35. Moreover, chronic dilatation of the STA has occasionally been observed after bypass surgery performed in MMD with the aim of providing more blood flow 40. A typical case of MMD treated with STA-MCA bypass is described in Figure 3.

#### (ii) Chronic ICA and MCA insufficiency

Theoretically, STA-MCA bypass can be used to treat symptomatic atherosclerotic disease of the ICA and MCA 41. Unfortunately, a trial published in the New England Journal of Medicine in 1985 and the Carotid Occlusion Surgery Study (COSS) which was published in JAMA in 2011, showed that EC-IC (mainly STA-MCA and OA-MCA to a lesser degree) bypass provided no benefit in cases of atherosclerotic narrowing or occlusion of the ipsilateral ICA or MCA 42, 43.

However, the trial published in the New England Journal of Medicine failed to stratify patients by risk to determine which would receive the greatest benefit from this intervention 42. Additionally, the COSS trial, which was published in JAMA, did not specifically investigate patients in whom the best medical therapy failed 43. In addition, many researchers believe that the blood flow provided by STA-MCA bypass would be insufficient for ICA or some MCA occlusions 44-46.

Therefore, theoretically, some carefully selected patients could still benefit from STA-MCA bypass 47, 48. Recently, it has been proposed that in some patients in whom optimal medical therapy fails or in whom flow-limiting stenosis is observed on a perfusion-dependent neurological examination, STA-MCA bypass could represent an effective and safe option as a rescue therapy 44, 49. Therefore, in these patients, STA-MCA bypass is promising.

In addition, in chronic ICA and MCA insufficiency, these bypasses increase blood flow, such as the STA trunk to MCA bypass with short radial artery or STA-MCA double anastomoses, and may also be effective for providing neurological improvement in symptomatic atherosclerotic disease of the ICA and MCA 50, 51. However, this hypothesis remains to be explored.

#### (iii) Acute ischemic stroke

In acute ischemic stroke (AIS) resulting from atherosclerotic occlusion of the ICA or MCA, the role of STA-MCA bypass remains poorly understood; for example, it remains unknown whether STA-MCA bypass provide any beneficial effects in affected patients 52. Attempts at STA-MCA bypass in AIS have been continuing, even though this procedure is considered controversial 52-54.

Recently, Rice et al. (2018) performed a study of a large series and found that STA-MCA bypass, when performed in a setting of symptomatic AIS within 1 week, may confer a higher risk of perioperative stroke, including progression of ischemia or hemorrhagic transformation. Patients undergoing urgent bypass for unstable stroke symptoms might have the highest risk for perioperative stroke 52.

However, Hwang et al. (2011) found that STA-MCA bypass may be beneficial in AIS or stroke showing progress of a small infarction 55. Lee et al. (2013) obtained a result similar to that of Hwang et al. 54. In 2017, Park et al. proposed the following MRI-related inclusion criteria for urgent STA-MCA bypass: acute infarct volume <70 ml with a ratio of perfusion/diffusion lesion volume ≥1.2 and a regional cerebral blood volume ratio >0.85 56.

Therefore, for STA-MCA bypass performed in AIS, the mainstream belief is that acutely symptomatic patients with ICA or MCA occlusion who continue to have recurrent ischemic symptoms may, in a very few selected cases, be indicated for a bypass, although a low-flow procedure is a far better bypass than a high-flow procedure in the setting of an acute stroke when the aim is to limit hemorrhagic complications 56-58.

### (3). Bypass for intracranial complex aneurysms

Complex aneurysms include large aneurysms, those with involvement of perforators, those from which branch arteries originate, or those that represent refractory lesions 59, 60. In treating these complex aneurysms, cerebral revascularization remains a major option that can serve as flow replacement bypass during aneurysmal trapping or insurance bypass during temporary parent artery occlusion 61.

In cerebral revascularizations, STA could serve as a donor vessel for low flow bypasses, which include STA-MCA, STA-anterior cerebral artery (ACA), and STA-superior cerebellar artery (SCA). In STA-ACA bypass, of note, an interposition graft may be needed for the long anatomical distance between STA and ACA. In case of high flow bypass between flow internal maxillary artery (IMA) and MCA, ACA, PCA, or ICA, STA can be used as an interposition vessel. 62-66. In addition, the STA can also serve as an interposition graft in IC-IC bypass after aneurysm resection 67-71.

STA-MCA bypass is most suitable for complex MCA and ICA aneurysms when the recipient is the M3/M4 segment 61, 72. To induce intra-aneurysmal thrombus and avoid distal ischemia, STA-MCA can be performed during proximal/distal clipping of the parent artery 61, 73. Insurance STA-MCA bypass has been performed to support the MCA territory during its prolonged temporary occlusion or during installation of high-flow bypass 61.

STA-MCA bypass is the standard mode of cerebral revascularization in complex MCA and ICA aneurysms. However, STA-MCA bypass provides a low-flow system, and insufficient blood flow may result in negative events following therapeutic occlusion of the main trunk arteries that should be replaced by high-flow revascularization 74.

The internal maxillary artery (IMA) is suitable to provide sufficient blood flow, and the STA trunk graft can be used for bypass of the IMA to proximal MCA in complex aneurysms 75-78. Recently, IMA bypass was redefined as a new “workhorse” to replace conventional cervical artery bypass in the field of high-flow bypass 74, 79.

**Figure 4:**
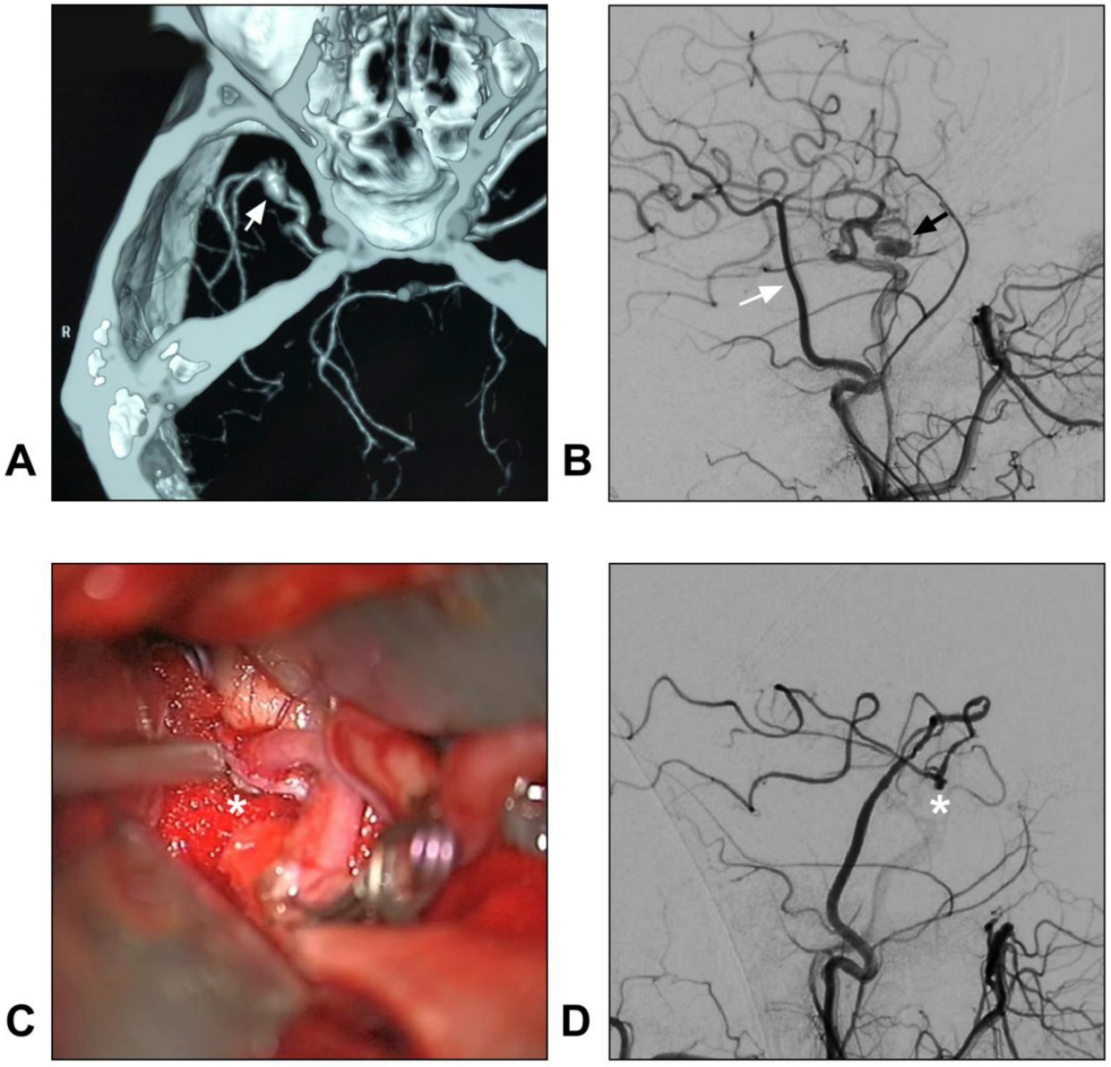
** STA-MCA bypass for intracranial aneurysm.** A: CTA reveals a dissecting aneurysm on the trunk of the MCA (arrow); B: DSA of the CCA shows the STA (white arrow) and aneurysm (black arrow); C: An end-to-side anastomosis between the STA and MCA trunk (asterisk) is performed; D: Postoperative angiogram of the ECA shows the STA-MCA bypass (asterisk) is patent. **Abbreviations: C**CA: common carotid artery; CTA: computed tomography angiography; DSA: digital subtraction angiography; ECA: external carotid artery; MCA: middle cerebral artery; STA: superficial temporal artery.

In addition, with a larger caliber, the STA trunk can provide more blood flow than the distal STA branches do. Hence, STA trunk-to-M2 of MCA or proximal PCA high-flow bypass with a short radial artery interposition graft should not be forgotten as it represents an excellent bypass to add to the armamentarium of choices when considering bypass options for complex aneurysms 80, 81. A typical case of an aneurysm treated with STA-MCA bypass is described in Figure 4.

### (4). Dural arteriovenous fistula

DAVF is an arteriovenous shunt located in the dural wall. The meningeal branches of the ECA and ICA are the main feeding arteries in DAVFs, with the middle meningeal artery (MMA) and OA the most commonly involved 82-84. Occasionally, the STA can be involved in a DAVF. The location of fistula point in STA supplied DAVFs could be at the superior sagittal sinus, the transverse-sigmoid sinus, the anterior cranial fossa, or the tentorial region 6, 84-89. When the STA serves as the main feeding artery, it can become swollen and thick 90, 91.

**Figure 5:**
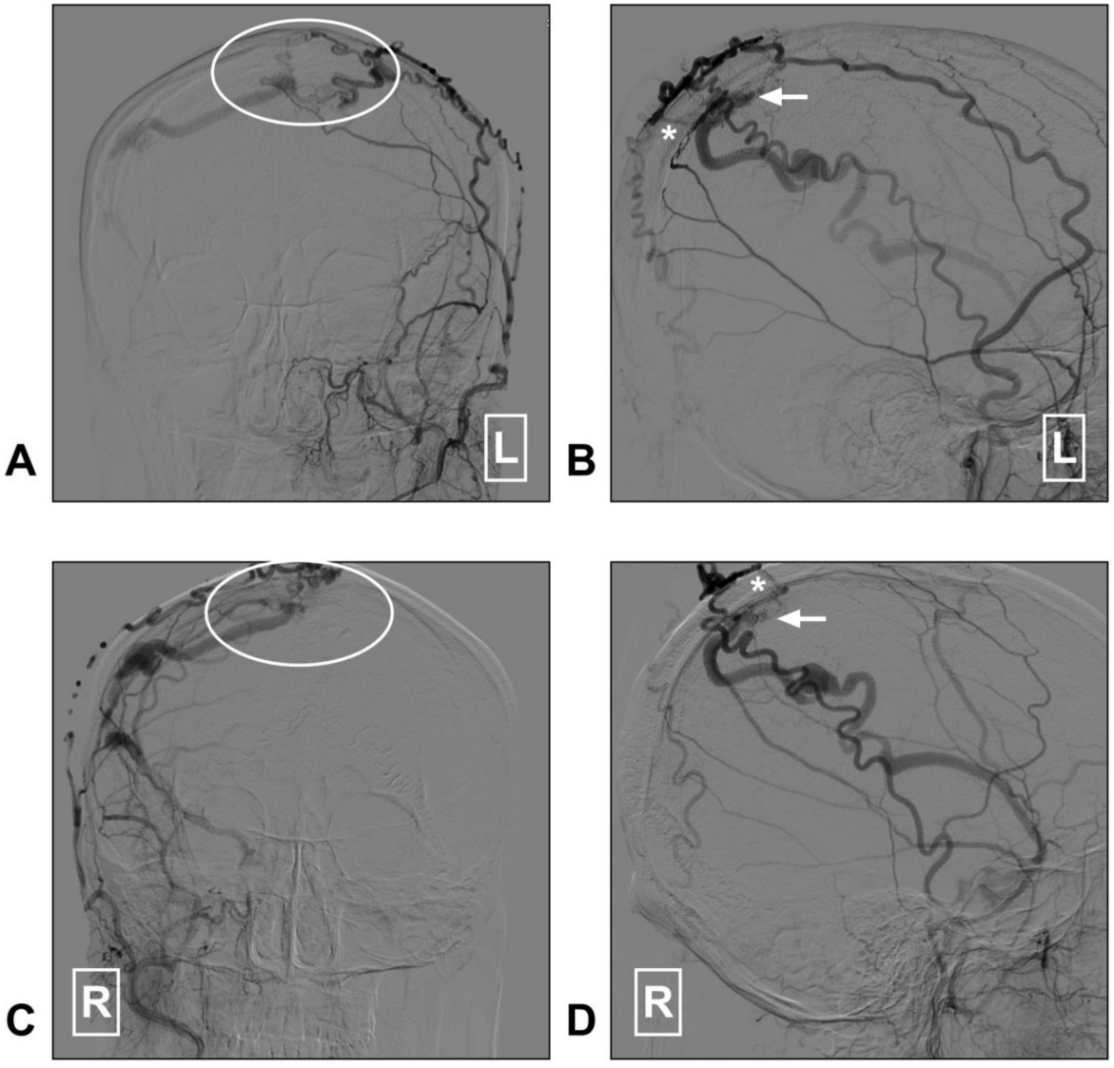
** STA supplied superior sagittal sinus DAVF.** A: Angiogram of the left ECA in AP view shows a superior sagittal sinus DAVF (white ellipse); B: Angiogram of the left ECA in lateral view shows the transosseous branches (asterisk) of the STA feed the DAVF. The arrow indicates the fistula point. C: Angiogram of the right ECA in AP view shows a superior sagittal sinus DAVF (white ellipse); D: Angiogram of the right ECA in lateral view indicates the fistula point (arrow). The transosseous branch (asterisk) of the right STA also feeds the DAVF. **Abbreviation:** AP: anterioposterior; DAVF: dural arteriovenous fistula; ECA: external carotid artery; L: right; R: right; STA: superficial temporal artery.

Currently, in DAVF embolization, the STA is known to be poorly suitable because a pressure gradient of its transosseous branches may limit embolic agent penetration toward the shunt point 92, 93. Recently, dual-lumen balloon has been proposed as a useful tool that may help to facilitate the penetration of liquid embolic agents from the STA 94. However, DAVF embolization via the STA is not a completely safe path and it carries the risk of cast extrusion 95. A DAVF case with STA as the feeding artery is described in Figure 5.

### (5). Intraoperative angiography and EVT path

In neurovascular diseases, intraoperative angiography is very useful for evaluating surgical clipping/excision and determining whether any residual lesion remains 96. Seldinger technique, in which a catheter is advanced into the ICA or ECA through the femoral artery, is a standard method, but performing intraoperative angiography through the femoral artery is sometimes not convenient during a craniotomy 97.

The STA is a good path for intraoperative angiography 98. STA puncture and retrograde advancement of the catheter down through the STA to the level of the carotid bifurcation could achieve intracranial angiography 7. In order to preserve the STA main trunk after angiographic procedure, the division of the STA can be selected for the cannulation site 99.

Finally, the STA can be used as the path to perform EVTs 100, 101. For instance, after the STA is exposed, incised and cannulated, a maxillary AVM can be embolized via the STA 102. Alternatively, in selected cases, the STA may represent a valid alternative approach for performing an EVT in a short common carotid artery dissection 103.

## Conclusion

This PRISMA-compliant systematic review of the clinical importance of STA in neurovascular diseases shows that the STA is one of the terminal branches of the external carotid artery and that it gives off many branches that supply the scalp and muscles. STA-MCA bypass is very useful for intracranial ischemic diseases. For intracranial complex aneurysms, STA bypass remains a major option that can serve as flow replacement bypass during aneurysmal trapping or insurance bypass during temporary parent artery occlusion. Occasionally, the STA can be involved in DAVFs via its transosseous branches. In addition, the STA can be used as a path for intraoperative angiography or performing an EVT procedure. Therefore, the STA is a very important artery in neurovascular diseases.
